# *Lactobacillus plantarum 299v* Reduces the Incidence of *Clostridium difficile* Infection in Nephrology and Transplantation Ward—Results of One Year Extended Study

**DOI:** 10.3390/nu10111574

**Published:** 2018-10-24

**Authors:** Sylwia Dudzicz, Agata Kujawa-Szewieczek, Katarzyna Kwiecień, Andrzej Więcek, Marcin Adamczak

**Affiliations:** Department of Nephrology, Transplantation and Internal Medicine, Medical University of Silesia in Katowice, Francuska 20/24, 40-027 Katowice, Poland; sylwia.dudzicz@gmail.com (S.D.); agata.szewieczek@gmail.com (A.K.-S.); kapril@o2.pl (K.K.); awiecek@sum.edu.pl (A.W.)

**Keywords:** *Clostridium difficile* infection, *Lactobacillus plantarum 299v*, organ transplantation, nephrology and transplantation ward

## Abstract

**Background:***Lactobacillus plantarum 299v* (LP299v) is a probiotic strain which influences on the intestinal bacterial flora. This is why, it has been introduced into clinical practice for the prevention and treatment of diarrheal disorders and alleviation of their symptoms in patients during antibiotic therapy. However, the use of probiotics in the prophylaxis of *Clostridium difficile* infections (CDI) in these patients is problematic. The aim of this clinical, retrospective, single-centre study was to analyse the incidence of CDI among patients hospitalized in the nephrology and transplantation ward in the period before, during and after stopping of LP299v prophylaxis. **Methods:** Among 5341 patients hospitalized in the nephrology and transplantation ward over a three year period, 34 patients with CDI were diagnosed and included in this analysis. From December 2013 to December 2014 all patients under antibiotic and immunosuppressive therapies received LP299v as a prophylaxis of CDI. The observation period consisted of three twelve-months periods: before, during LP299v use and after stopping of such method of CDI prevention. **Results:** A significant (*p* = 0.0003) reduction of CDI incidence during LP299v use (0.11%) was observed compared to two other periods, that is, before and after LP299v use (1.03% and 0.77%, respectively). **Conclusions:** Routine use of LP299v as a CDI prophylaxis may prevent CDI during antibiotics therapy in patients treated with immunosuppressive agents in nephrology and transplantation ward.

## 1. Introduction

During the last years infection with *Clostridium difficile* was one of the most common causes of antibiotic-associated diarrhoea and nosocomial diarrhoea [1]. In last decades increase incidence of *Clostridium difficile* infection (CDI) and mortality from CDI in a global scale were observed. There was also a higher incidence of severe, complicated infections or CDI recurrence [2,3,4]. During recent years, an increase prevalence of hypervirulent endemic strain of *Clostridium difficile* ribotype 27 has been observed. In patients infected by this strain a more frequent complicated or severe CDI forms are observed. Moreover, an increase mortality and frequent recurrences infection with CDI infection has also been observed [5,6]. Most susceptible subjects to CDI are patients after solid organ transplantation and patients receiving immunosuppressive drugs due to any other reason [7,8,9]. In relation to observed worldwide increase number of infection and higher mortality from CDI the method of prophylaxis of *Clostridium difficile* infections seems to be crucial, especially in ward with hospitalized such patients.

So far, the efficacy of probiotics in the prevention of *Clostridium difficile* infection in group of patients undergoing antibiotic therapy has not been proven. In a meta-analysis of 39 randomized controlled trials including 9955 patients, Goldenberg et al. showed that the co-administration of probiotics and antibiotics was associated with a lower risk for CDI versus placebo or no treatment (1.5% vs. 4.0%; *p* < 0.001; relative risk—RR = 0.40; 95% confidence interval—CI 0.30–0.52) [10]. In the meta-analysis of 18 trials which consisted of data from 6851 participants, Johnston et al. found that probiotics reduced the risk of CDI by 66%. They also observed, that such prevention is a very useful among patients treated with 2 or more antibiotics [11]. In a largest up to date randomized, clinical study in this area (*n* = 2889, PLACIDE study) it has not been proved that the multistrain formulation of *lactobacilli* and *bifidobacteria* may decrease the risk of CDI [12]. Nevertheless, it should be noted that the efficacy of *Lactobacillus plantarum 299v* (LP299v) was not evaluated in this study. Moreover, in the majority of studies, included in above mentioned meta-analyses, as well as, in PLACIDE study, patients under immunosuppressive therapy were excluded.

The *Lactobacillus plantarum 299v* (LP299v) is lactic acid bacteria from Gram-positive bacteria group that naturally occurs on the surface of human intestinal mucosa [13]. Specific properties to the gut mucosa colonization are associated with mannose-dependent mechanism of adhesion of LP299v to the human intestinal epithelium. Other LP299v surface adhesins and glycolytic enzymes include glyceraldehyde 3-phosphate dehydrogenase (GAPDH), enolase (ENO) and phosphoglycerate kinase (PGK). GAPDH blocks adhesion of streptococci group A bacteria, *Staphylococci*, *Candida albicans* and *Schistosoma mansoni*. ENO inhibits adhesion of *Streptococcus pneumoniae*, *Streptococcus aureus* and *Candida albicans* [14,15,16]. The unique properties of this bacterial strain prevent adhesion of many pathogens to intestinal epithelium and reduces of bacterial translocation from the intestinal lumen into the blood [17,18,19,20,21,22]. This strain also regulates the inflammatory response of intestinal mucosa epithelium [19]. The strain 299v of *Lactobacillus plantarum* has been found to decrease the severity of gastrointestinal symptoms during antibiotic therapy [23].

The data about the efficacy of LP299v for preventing *Clostridium difficile* infections are very limited.

The aim of this retrospective study was to analyse the rate of infections with *Clostridium difficile* before, during and after cessation of routine LP299v prophylaxis, as a prevention of CDI among hospitalized patients in nephrology and transplantation ward. The above-mentioned periods lasted twelve months.

## 2. Material and Methods

The current study is the continuation of the already published results from our study [23], with the extended by one year observation period.

Among 5341 patients hospitalized in the Department of Nephrology, Transplantation and Internal Medicine, Medical University of Silesia in Katowice (Poland) during a three-year period (1 October 2012–1 October 2013, 1 December 2013–1 December 2014 and 1 February 2015–1 February 2016), 34 patients with non-community acquired CDI were diagnosed and enrolled into this retrospective, observational single-centre study. Among them, 24 CDI patients were from high risk group of patients that is, patients during immunosuppression therapy. Community-acquired CDI cases were excluded from the current analysis.

In this Department, all patients who underwent antibiotic therapy routinely received probiotics formulations as a prevention of antibiotic-associated diarrhoea or *Clostridium difficile* infection. Between 1 October 2012 and 1 October 2013 as well as 1 February 2015 and 1 February 2016 the most commonly used probiotics preparations were those containing the following bacterial strains: *Bifidobacterium lactis*, *Lactobacillus acidophilus*, *Lactobacillus delbrueckii*, *Lactobacillus rhamnosus* and *Saccharomyces boulardii.* Between 1 December 2013 and 1 December 2014, the routine administration of LP299v as a prophylaxis of *Clostridium difficile* infection was initiated in all patients under antibiotics therapy and immunosuppressive therapy. For further analysis, the observation period was divided into three twelve-months intervals before (1 October 2012 to 1 October 2013), during (1 December 2013 to 1 December 2014) and after cessation of LP299v use (1 February 2015 to 1 February 2016). In these periods 1742, 1791 and 1808 patients were hospitalized, respectively. Among them 267, 277 and 294 patients were at high risk CDI that is, during antibiotics and immunosuppressive therapy, respectively.

From December 2013 to December 2014 all patients on antibiotics therapy and receiving immunosuppressive drugs (i.e., 277 patients) were enrolled to LP299v probiotic prophylaxis. The probiotic was administered orally at a daily dose of one capsule (Sanprobi IBS^®^ Sanprobi Sp. z o.o., Sp. k., Szczecin, Poland; capsules manufacturer—Institute Rosell-Lallemand, Montreal, Canada; LP299v strain owner-Probi AB, Lund, Sweden) for the entire antibiotic therapy period. One capsule of Sanprobi IBS^®^ contains at least 10 × 10^9^ colony forming units (CFU) of LP299v. This prophylaxis was ceased in 1 December 2014.

Diagnostics of CDI were carried out in accordance to the guidelines of the European Society of Clinical Microbiology and Infectious Diseases (ESCMID) [24]. For this purpose, a two-step diagnostic algorithm was used. Firstly, detection of the *Clostridium difficile* toxin in stool samples from patients with diarrhoea using the enzyme immunoassay Premier^®^ Toxins A&B test (Meridian Bioscience, Inc., Cincinnati, OH, USA) was performed. In the case of a negative test result, the second step was to bacterial culture stool samples using the toxigenic culture with chromID^®^ *Clostridium difficile* culture media (bioMérieux S.A., Marcy L’Etoile, France) under anaerobic conditions. After 48 h, obtained bacterial colonies was cultured on selective media and were tested for toxin production once again with enzyme immunoassay Premier^®^ Toxins A&B test (Meridian Bioscience, Inc., Cincinnati, OH, USA).

Severe CDI are characterized by: community-acquired CDI requiring hospitalization, occurrence of *megacolon toxicum* or perforation which may require colectomy, necessity of hospitalization in intensive care unit or death due to CDI within 30 days from onset of the symptoms. The risk factors for severe infection included in this study are: age >70 years, ileus, suspicion of CDI in computer tomography, leucocytosis >20 G/L, serum creatinine concentration >180 µmol/L, serum albumin concentration <2.5 g/L and previous surgery within the last 30 days.

Recurrent CDI was diagnosed in the case of re-appearance of the infection symptoms before up to two months from the end of last CDI episode.

In case of onset of the symptoms after 48 h of hospital admission nosocomial infection was diagnosed. In case of onset of symptoms up to 48 h of hospital admission or positive history of healthcare exposure within more than the last three months community-acquired CDI was diagnosed.

STATISTICA 12.0 PL for Windows software package (StatSoft Polska, Kraków, Poland) was used for statistical analysis. To evaluated the statistical significance of differences between the results, the chi^2^ test was used. The *p*-value below 0.05 was considered as statistically significant. The results are presented as mean values with standard deviations.

## 3. Results

During the entire 3 years period 34 non-community acquired CDI cases were diagnosed. In this period 6 community-acquired cases were diagnosed. Such cases were not analysed. The diagnosis of CDI was based on a positive result of toxin detection in a stool sample among 31 patients with clinical symptoms of this infection. In 3 cases, with negative toxin assay result, the toxigenic culture test was positive. The characteristics of patients with CDI (*n* = 34) are shown in Table 1.

Among these patients were 12 (35%) women and 22 (65%) were men. The mean age of CDI patients was 57 ± 15 years. Depending on the age range, the infection was present in patients aged below 40, between 40 and 50 and above 50 years of age in 4 (12%), 6 (18%) and 24 (70%) patients, respectively.

The mean time from the onset of symptoms to the CDI diagnosis was 5.0 ± 6.1 days. The main symptom occurring in 34 (100%) patients was diarrhoea of varying intensity. The mean duration of diarrhoea was 9.1 ± 7.3 days and the average number of stools per a day was 5.4 ± 2.5. Among the additional symptoms fever, abdominal pain and vomiting occurred in in 19 (56%), 15 (44%) and 1 (3%) patients, respectively.

Nosocomial infection was diagnosed in 24 patients (71%) of total CDI group. In 10 cases (29%), the infection might be associated with health care facilities. In 28 (82%) cases of CDI, patients were hospitalized at least once during the three months before the appearance of CDI symptoms. Seventeen (50%) patients were hospitalized more than once.

Thirty (88%) out of 34 patients with *Clostridium difficile* infection reports a history of antibiotic therapy in the four weeks before appearance CDI symptoms. Moreover, 23 (68%) out of these patients were treated with more than one antibiotic. The time from the beginning of antibiotic therapy to the onset of clinical symptoms of CDI was 9.8 ± 5.7 days. Among CDI patients the common reason of antibiotics use was urinary tract infection. The most frequently used antibiotics were: fluoroquinolones, carbapenems, monobactams and penicillins. The indications for antibiotic use in the CDI group and the antibiotic exposure in this group of patients are presented in Table 2.

Histamine 2 receptor blockers and proton pump inhibitors were used in 3 (9%) and 28 (82%) patients respectively from the group of patients with CDI.

Severe CDI was diagnosed in three (9.1%) patients and two of them were after solid organ transplantation. Two of these patients with severe CDI died within 30 days after the appearance of CDI symptoms.

During the observation period before the initiation of LP299v use (October 2012–October 2013) CDI was diagnosed in 18 patients. After implementation of LP299v strain as prevention of *Clostridium difficile* infection number of CDI cases was reduced to two per year (December 2013–December 2014). After cessation of this prophylaxis (February 2015–February 2016) incidence of CDI increased to 14 cases. As shown in Table 3, after starting of LP299v prophylaxis the incidence rate of CDI significantly declined from 10.3 to 1.1 per 1000 patients hospitalized (RR 0.11; CI 0.03–0.47; *p* = 0.0003). After cessation of use the above-mentioned prevention incidence of CDI significantly increased from 1.1 to 7.7 per 1000 hospitalized patients (RR 6.93; CI 1.58–30.47; *p* = 0.0028) (Table 3).

The clinical characteristic of CDI patients in different analysed periods are presented in Table 4.

Among the patients on immunosuppressive therapy during the three years period 24 CDI cases were diagnosed. During the observation period before the implementation of LP299v (October 2012–October 2013) CDI was diagnosed in 12 such patients. After introduction of LP299v strain as prevention of *Clostridium difficile* infection number of CDI cases was reduced to two per year (between December 2013 and December 2014). After cessation of this prophylaxis (between February 2015 and February 2016) incidence of CDI increased to 10 cases in this group. In patients during immunosuppressive therapy after start of LP299v prophylaxis the incidence rate of CDI significantly declined from 44.9 to 7.2 per 1000 high risk patients (RR 0.16; CI 0.02–0.71; *p* = 0.005) and after cessation of use the above-mentioned prevention incidence of CDI significantly increased from 7.2 to 34 per 1000 high risk patients (RR 4.71; CI 1.04–21.31; *p* = 0.025) (Table 3).

It should be mentioned that 10 of all CDI patients was not treated with immunosuppressive therapy, therefore these patients did not received prophylaxis with the LP299v. During the observation period before the introduction of LP299v CDI was diagnosed in 6 such patients, after implementation of LP299v strain as prevention of Clostridium difficile infection number of CDI cases was reduced to 0 per year and after cessation of this prophylaxis incidence of CDI increased to 4 cases in this group. After the introduction of LP299v prophylaxis the incidence rate of CDI significantly declined from 4.08 to 0 per 1000 high risk patients (RR 0.075; CI 0.004–1.329; *p* = 0.013) and after cessation of CDI prevention significantly increased from 0 to 2.65 per 1000 high risk patients (RR 9.0; CI 0.485–167.03; *p* = 0.045).

It should be noted, that there have been no cases of severe CDI during the period of LP299v prophylaxis. The analysis of severe CDI and selective risk factors of severe CDI in different observation periods are shown in Table 5 and Table 6, respectively.

In mentioned three twelve-months observational periods the recurrence of *Clostridium difficile* infection was diagnosed in five patients. In two of them the CDI recurrence was recognized after initiation of the LP299v implementation. Nevertheless, in these two cases gastrointestinal symptoms and the severity of the infection (duration of diarrhoea, number of stools per a day, average CRP serum concentrations) was milder during this method of prophylaxis (Table 7).

Two hundred and seventy-seven patients treated with antibiotics therapy and on immunosuppression hospitalized in the period between December 2013 and December 2014 were given LP299v as a CDI prophylaxis. It is calculated that such a LP299v prophylaxis should be used in 15 patients to prevent the occurrence of CDI in one patient hospitalized in this ward (number needed to treat: 15). The average time of prophylaxis duration in our observation was 14 ± 7 days. The cost of this CDI prevention method was 17.5 PLN (4.1 €) per one patient for the entire prophylaxis period. Therefore, the cost of one CDI case prevention is 262.5 PLN (61.5 €).

## 4. Discussion

In the current study, we have assessed the effect of *Lactobacillus plantarum 299v* use as a prophylaxis of *Clostridium difficile* infection in patients hospitalized in nephrology and transplantation ward. It was observed a significant decrease of the CDI incidence after administration of LP299v use and subsequently significant increase after cessation of the above-mentioned prophylaxis. The above-mentioned periods lasted twelve months. In addition, we did not notice severe CDI episodes after introduction of LP299v prophylaxis. Results presented in the current paper are the continuation of the already published results from our study [23], with the extended by one year observation period.

Shen et al. [25] in a meta-analysis of 19 studies including 6261 patients during antibiotic therapy found that in patients whom receiving probiotics, the CDI risk was lower in compare to the control group (1.6% vs. 3.9%; *p* < 0.001). Moreover, it was shown that the effectiveness of probiotics was greater if they started to be administered with the beginning of antibiotic therapy and every day of delay caused decrease of their efficiency. The probiotic used during 2 days of starting antibiotic therapy caused a significant reduction in CDI incidence than a later administration. In the current study, we introduce LP299v therapy in the first day of antibiotics use.

Lönnermark et al. [26] in a double blind, placebo controlled trial investigated the effect of orally LP299v use on the incidence of antibiotic-associated diarrhoea. In this study patients received *Lactobacillus plantarum 299v* in a dose 10 × 10^9^ per a day. The authors did not observe significant difference in toxin-producing *Clostridium difficile* carriage rate between the study and control group. However, in patients receiving LP299 reduction of the incidence of loose stools and nausea were observed [26]. In current study, it was also documented the shorter duration of diarrhoea, decreased number of stools and lower serum C-reactive protein concentrations in patients with CDI during LP299v prophylaxis.

Klarin et al. [27] evaluated the *Clostridium difficile* colonization in a group of 44 patients under antibiotic therapy and hospitalized in the intensive care unit. *Clostridium difficile* colonization was reported in 4 cases (19%) in the control group (*n* = 21) while no toxins in the faecal samples were found in patients receiving LP299v. These results demonstrated that the use of LP299v in patients undergoing antibiotic therapy and with high risk of CDI may prevent the *Clostridium difficile* colonization in the gut [27]. In current study, there was no possibility of analysis the rate of patient colonization by *Clostridium difficile* after the introduction of LP299v prophylaxis due to its retrospective character.

In a double blind, placebo-controlled study Wullt et al. [28] analysed the ability of LP299v to prevent CDI recurrence. Twenty-one patients were included in this study. Studied patients were enrolled into two groups: receiving metronidazole with LP299v and receiving metronidazole with placebo. There was a tendency to lower incidence of CDI recurrences until 70 days after treatment in the LP299v use group compared to the control group. In current study, the symptoms of infection during CDI recurrence were milder. An additional interesting aspect, analysed was by Johansson et al. [29], is the relationship between LP299v treatment and increased concentration of short-chain fatty acids (SCFAs) in faecal samples from healthy volunteers. SCFAs may inhibit growth of certain microorganisms [30,31] and they are important source of energy for the colonic mucosal cells [32]. Positive correlation between SCFA production, water and sodium absorption in the colon and reduction of diarrhoeal symptoms has been observed in other studies [29]. Wullt et al. [33] studied the effect of LP299v intake on the SCFAs concentration in stool samples from patients treated with metronidazole because of the CDI recurrence. They found that LP299v use may reduce the negative effect of antibiotics on the fermentation process in the colon [33].

Rayes et al., in a prospective, randomized placebo-controlled study, assessed the occurrence of postoperative infections in patients after liver transplantation divided into three groups differing with the way supplied with early enteral nutrition. Significantly more infections occurred in patients with selective bowel decontamination (SBD) that is, therapy with 80 mg of tobramycin, 500 mg of amphotericin B and 100 mg colistin sulphate compared with patients receiving LP299 and oat fibre. The incidence of infections in placebo group (heat-killed LP299 and oat fibre) was also lower than in patients with SBD [34].

Probiotics implementation as a prevention of infection during post-operative period after liver transplantation presented Grąt et al. [35]. Patients receiving probiotics (*Bifidobacterium bifidum*, *Lactobacillus acidophilus*, *Lactobacillus casei* and *Lactococcus lactis*) had significantly reduced infection rates compared to the placebo group. In addition, it was noted in the group treated with probiotics the improvement of early biochemical parameters of graft function that is, lower bilirubin concentration and faster decrease of aspartate and alanine aminotransferase activities.

It should be stressed that diarrhoea in patients after organ transplantation has important clinical meaning. It may lead to the reduction of immunosuppressive agent blood concentration which can lead to development of acute rejection [36]. Moreover, in the case of kidney transplantation dehydration may lead to acute kidney injury.

Additionally, modulation of the intestinal microbiota with LP299v may decrease the risk of intestinal mucosal injury and appearance of gastrointestinal symptoms caused by proton pump inhibitors frequently used in group of patients treated with glucocorticoids as a part of immunosuppressive therapy [37].

One of the effective methods of treatment of recurrence CDI is faecal microbiota transplantation (FMT) [38,39]. The effectiveness of this method is high but studies concerning these issues mainly included immunocompetent patients. There are only very few case studies on FMT among patients on immunosuppression. Friedman-Moraco et al. [40] described 2 cases of patients after solid organ transplantation with recurrence CDI treated with FMT with good results. It should be stressed that there are no enough data concerning bacteriological and immunological safety of FMT in patients treated with immunosuppressive agents and/or after solid organ transplantation [38].

There are several studies analysing the impact of patient-to-patient contact on the increased risk of CDI. A patient who is a roommate of CDI patient has an increased risk of this infection [41]. Therefore, it may suggest that, in the current study, also the hospitalized patients not treated with LP299v (patients not on immunosuppression) may profit with CDI prophylaxis that is, LP299v use in the patients on immunosuppression.

An important aspect related to the increased incidence of nosocomial CDI is a significant increase of the hospitalization costs. Based on the result of the current study it is calculated that the cost of CDI single case prevention with the LP299v use is 262.5 PLN (61.5 €). Such good pharmacoeconomic properties of LP299v preventive use are due to low cost of LP299v (1.25 PLN/0.3 € per dose; 17.5 PLN/4.1 € per patients undergoing prevention) and low number needed to treat (i.e., 15 patients). The total annual cost related to CDI in European Union in 2006 was estimated at €3 billion and is estimated at over €4 billion in 2015 [42]. The estimated costs of individual CDI case vary, from $8911 to $30,049 [42,43]. Therefore, it is suggested that it is several magnitudes higher than the cost of LP299v preventive use. The effective prevention of CDI with the LP299v use would significantly reduce the financial outlay for the treatment of CDI [42,43].

Our study has some limitations. The most important is the lack of placebo groups. This is due to retrospective, observational design of the current study. The results achieved in the current study, concerning the efficacy of LP299v as a prophylaxis CDI measure should be undoubtedly proven in a placebo-controlled randomized clinical trial.

In summary in the current study we have demonstrated that routine use of *Lactobacillus plantarum 299v* as a prophylaxis during antibiotics therapy in patients treated with immunosuppressive drugs in nephrology and transplantation ward may prevent *Clostridium difficile* infection.

## Figures and Tables

**Table 1:** Clinical characteristics of patients diagnosed with *Clostridium difficile* infections (CDI) (*n* = 34).

	CDI Patients (*n* = 34)
Age (years)	57 ± 15
Gender (M/F)	22/12
BMI (kg/m^2^)	25 ± 4
Diabetes mellitus (*n*)	13
Chronic Kidney Disease (*n*)	21
Dialysis (*n*)	3
Systemic vasculitis (*n*)	3
Liver cirrhosis (*n*)	4
Inflammatory Bowel Disease (*n*)	0
Cancer (*n*)	4
Patients after organ transplantation (*n*)	18

**Table 2:** Cause of antibiotic therapy and type of antibiotic exposure among CDI patients before the CDI onset.

The Cause for Antibiotic Therapy	*n*
Urinary tract infection	18
Pneumonia	1
Upper respiratory tract infection	1
Peritoneal dialysis-related infection	2
Other	7
**Type of Antibiotic Exposure**	
Fluoroquinolones	15
Carbapenems and monobactams	15
Penicillins	11
Cephalosporins	8
Aminoglycosides	3
Cotrimoxazole	3
Others	3

**Table 3:** The number of CDI cases and incidence before, during and after cessation of LP299v administration as a routine prophylaxis in the nephrology and transplantation ward among all patients and patients during immunosuppressive therapy hospitalized in the nephrology and transplantation ward.

	All Hospitalized Patients		Patients on Immunosuppressive Therapy	
	(*n*)	(%)	(*n*)	(%)
Before introduction of LP299v	18	1.03% ***	12	4.49% **
During prophylaxis of LP299v	2	0.11%	2	0.72%
After cessation of LP299v	14	0.77% **	10	3.4% *

* *p* < 0.05, ** *p* < 0.01, *** *p* < 0.001 vs. incidence during prophylaxis of LP299v.

**Table 4:** Clinical characteristics of patients with *Clostridium difficile* infection (CDI) in periods before, during and after cessation of LP299v administration (*n* = 34). The above-mentioned periods lasted twelve months.

	Before LP299v Use (*n* = 18)	During LP299v Use (*n* = 2)	After Cessation of LP299v (*n* = 14)
Age (years)	57 ± 15	53 ± 16	55 ± 15
Gender (M/F)	9/9	2/0	11/3
BMI (kg/m^2^)	24 ± 4.5	23 ± 0	25 ± 3.7
Diabetes mellitus (*n*/%)	(6/33%)	(2/100%)	(5/36%)
Chronic Kidney Disease (*n*/%)	(13/72%)	(1/50%)	(7/50%)
Dialysis (*n*/%)	(1/6%)	(0/0%)	(2/14%)
Systemic vasculitis (*n*/%)	(2/11%)	(0/0%)	(1/7%)
Liver cirrhosis (*n*/%)	(3/17%)	(1/50%)	(0/0%)
Inflammatory Bowel Disease (*n*/%)	(0/0%)	(0/0%)	(0/0%)
Cancer (*n*/%)	(3/17%)	(0/0%)	(1/7%)
Patients after organ transplantation (*n*/%)	(8/44%)	(2/100%)	(8/57%)

**Table 5:** Severe *Clostridium difficile* infections in periods before, during and after cessation of LP299v administration (*n* = 3). The above-mentioned periods lasted twelve months.

	Before LP299v Use	During LP299v Use	After Cessation of LP299v Use
Megacolon toxicum (*n*)	0	0	0
Colectomy (*n*)	0	0	0
Transfer to the intensive care unit due to CDI complications (*n*)	1	0	0
Death (*n*)	1	0	1

**Table 6:** Risk factors of severe CDI in periods before, during and after cessation of LP299v administration (*n* = 34). The above-mentioned periods lasted twelve months.

	Before LP299v Use (*n*/%)	During LP299v Use (*n*/%)	After Cessation of LP299v Use (*n*/%)
Age > 70 years	4/22	0/0	1/7
WBC > 20 G/L	6/33	1/50	5/36
Serum creatinine > 180 µmol/l	13/72	0/0	9/64
Albumin < 2.5 g/dL	7/39	1/50	6/43
Bowel obstruction	0/0	0/0	0/0
Large intestine changes in computer tomography	1/6	1/50	2/14
Surgery in the last 30 days	4/22	1/50	5/36

**Table 7:** The analysis of the clinical features of the recurrence of CDI in two patients before and after initiation of LP299v prophylaxis. The above-mentioned periods lasted twelve months.

	Before LP299v Use	After LP299v Use
Duration of diarrhoea (days)	28 ± 37	9.5 ± 9
Number of stools per a day during CDI symptoms	8 ± 0	7 ± 0
Abdominal pain (*n*)	2	2
Vomiting (*n*)	0	0
Fever (*n*)	2	2
Average leucocytes count (G/L) during CDI symptoms	14.1 ± 9.9	14.4 ± 13.1
Average CRP serum concentrations (mg/L) during CDI symptoms	96.5 ± 34.7	43.8 ± 43.5
